# Novel models to improve access to medicines for chronic diseases in South Africa: an analysis of stakeholder perspectives on community-based distribution models

**DOI:** 10.1186/s40545-016-0082-6

**Published:** 2016-10-01

**Authors:** Bvudzai Priscilla Magadzire, Bruno Marchal, Kim Ward

**Affiliations:** 1 School of Public Health, University of the Western Cape, Private Bag X17, Bellville, 7535 South Africa; 2 Department of Public Health, Institute of Tropical Medicine, Antwerp, Belgium; 3 School of Pharmacy, University of the Western Cape, Bellville, South Africa

**Keywords:** Community-based distribution, Access to medicines, Pharmaceutical policy, South Africa

## Abstract

**Background:**

The rising demand for chronic disease treatment and the barriers to accessing these medicines have led to the development of novel models for distributing medicines in South Africa’s public sector, including distribution away from health centres, known as community-based distribution (CBD). In this article, we provide a typology of CBD models and outline perceived facilitators and barriers to their implementation using an adapted health systems framework with a view to analysing how future policy decisions on CBD could impact existing models and the health system as a whole.

**Methods:**

A qualitative exploratory study comprising in-depth interviews and non-participant observations was conducted between 2012 and 2014 in one province. Study participants consisted of frontline healthcare providers (HCPs) in the public sector and a few policy, supply chain and public health experts. Observations of processes occurred at two CBD sites. We conducted deductive analysis guided by the adapted framework.

**Results:**

Models varied in typology ranging from formal (approved by the Department of Health) to informal (demand-driven) and with or without user-fees. Processes and structures also differed, as did HCPs’ perceptions of what is appropriate. HCPs perceived that CBD models were largely *acceptable* to patients and *accommodating* of their needs. *Affordability* of services linked to charging of user-fees was a contested issue, requiring further exploration. CBD models operated in the absence of formal policy to guide implementation, and this, coupled with the involvement of non-health professionals, issues regarding medicines handling and storage; and limited patient counselling raised concerns about the quality of pharmaceutical services being delivered. Policy decisions on each of the health system elements will likely affect other elements and ultimately influence the structure and operational modalities of models. In anticipation of a future CBD policy, stakeholders cited the need for a c*ontext specific* lens in order to harmonise with current implementation efforts.

**Conclusion:**

A formal policy on CBD is required in an effort to standardise services for quality assurance purposes. Frontline HCPs should be involved in the development of such policy to ensure that existing arrangements already working well are not undermined. Further research will seek to contribute towards evidence-based development of policy and service delivery guidelines for CBD activities in South Africa.

## Background

South Africa shares with the rest of sub-Saharan Africa a high burden of chronic diseases, including HIV and non-communicable diseases [1]. This has led to an increasing demand for medicines for treatment of disease in a context of a weak health system [2]. The increased burden of disease has illuminated the need for the government to be more responsive to population needs and to ensure that people obtain health services (including accessing essential medicines) without suffering financial hardship. The latter are in line with principles of universal health coverage (UHC) [3].

The South African government released the National Health Insurance (NHI) White Paper in December 2015. This policy document discusses various health insurance modalities and reforms aimed at strengthening the country’s health system. These include: expanding access to pharmaceutical products, a primary healthcare re-engineering strategy and establishment of an office of health standards compliance. Furthermore, it describes a vision of what is required for the successful implementation of NHI [4].

Against this background, we have witnessed a shift in the local access to medicines (ATM) domain, from a largely health facility-based approach to providing medicines for chronic diseases to novel community-based distribution (CBD) models, also referred to as alternative distribution or out-of-clinic models [5]. While the term “distribution” within the broader medicine supply chain context encompasses ordering, transportation and logistics management at various levels [6], its use in this article is confined to logistics activities to get patient-ready pre-packaged medicines to patients. This has been referred by some authors as the “last mile”, where services are delivered to patients and often at the most vulnerable stage of distribution [7].

CBD models use community halls and similar gathering places as sites for medicine distribution, exploiting the proximity of these venues to patients’ homes. Sometimes, they also include home deliveries. These models are geared towards addressing various supply- and demand-side barriers to accessing medicines [8]. Such barriers include: long waiting times, overburdened health centres which discourage patients from collecting medicines and reducing travel costs to distant health facilities. Furthermore, CBD models can allow for task-shifting to mid-level cadres or even to expert patients in order to address human resource shortages [9, 10]. The latter is facilitated by the choice of target beneficiaries, i.e. stable patients not requiring regular contact with a healthcare provider (HCP). Such patients can be sufficiently empowered to self-manage [11] and have six-monthly consultations. CBD is not only recognised in South Africa as an interesting solution to restricted access to medicines [12, 13], but in many other developing countries, [14–16] including Mozambique [5, 17–19], Zambia [20] and Kenya [21]. CBD models are driven by non-governmental organisations (NGOs) in the majority of cases.

While CBD is gaining momentum in South Africa, the range of models and the pace of implementation are variable across provinces. This could be explained in part by the health system’s governance structure, which allow provinces a fair degree of autonomy in the administration of health services [22]. The Western Cape is one province where CBD has been widely implemented. In this province, CBD falls under the umbrella of community-based services, an important component of the broader primary healthcare (PHC) platform that features in the provincial strategy for health, Healthcare 2030 [23]. CBD is facilitated by centralised dispensing of patient-ready medicine packages by a private distributor to health facilities [24–27]. These packages can easily be transported to CBD points.

This article draws on selected findings from a broad exploratory study commissioned by the Western Cape Department of Health (WCDoH) to improve access to medicines (ATM). The overall study sought to identify strategies to address the challenge of missed appointments among patients with chronic diseases in the metropolitan district of Cape Town [24]. We also sought to understand the structure of ATM strategies and the facilitators and barriers to effective implementation. We targeted frontline healthcare providers (HCPs), most of whom engage with patients on a regular basis. These stakeholders have a critical role in the attainment of policy outcomes yet their role is often overlooked [28, 29]. Our research showed that many HCPs identified CBD (among a few others) as an existing innovative strategy for ensuring that medicines reach patients. However, they also cited challenges, of which the most important was the lack of policy to govern CBD activities even though their implementation was actually underway. The implication was that certain issues related to CBD could be open to multiple interpretations. We discovered early on that governing CBD activities was far from being simple, given that these are “non-traditional” mechanisms for medicines distribution.

As the development of a CBD policy is a current priority in South Africa, in this article we seek to contribute to the policy-making process by exploring how CBD models currently operate in the Western Cape Province’s local health system and identifying the perspectives of frontline HCPs regarding CBD models. In order to provide evidence that could inform policy design, we have adapted the health systems framework of van Olmen et al. (the framework) [30] as an analytical tool for the following reasons:

- a) Its ability to assist us to identify and discuss the key elements of CBD models (e.g. medicines supply, human resources, infrastructure and population) and to draw the interconnections between the elements which will be of relevance to the design of CBD policy;
- b) Its ability to frame CBD operations within the context of the broader health system;
- c) The importance it attaches to values and principles in policy-making [22].
- d) Its recognition of health systems as social systems which comprise people and organisations, and their interactions with others. As such, actors’ values, interests, norms and relationships also influence the ultimate character of the system [31].

In this article we use the framework to provide a systematic description of CBD models and to illustrate how the configuration of the elements in each CBD model contributes to its effectiveness. Finally, we explore how our findings could inform the development of an impending CBD policy by drawing upon the perspectives of stakeholders.

## Methods

### Study design

This exploratory qualitative study was conducted between 2012 and 2014 in the metropolitan district of Cape Town, which has the greatest proportion of patients and the greatest pressure on health services in the Western Cape Province [24, 25].

### Data collection

We used in-depth interviews, non-participant observations of two CBD sessions and document review as the data collection methods for this study.

#### Key informants

For this article, we drew from 45 in-depth interviews, which were conducted by the first author using a semi-structured interview guide. We purposively sampled informants who were most knowledgeable about the issues of interest from the following categories: (1) frontline HCPs, including doctors, nurses, pharmacists and pharmacist’s assistants (PAs) from four PHC facilities, (2) policy makers, (3) sub-district and provincial managers from the WCDoH, (4) private sector pharmacists, (5) academics with expertise in pharmaceutical policy and public health and (6) NGO staff (Table 1). Interviews were conducted in English and each interview lasted about one hour. All the interviews were conducted at a place convenient for the respondents, i.e. their place of work. Where possible, interviews were recorded; alternatively, notes were taken. Three participants refused to be recorded as a matter of preference. Once no information was generated from the interviews and saturation was reached, no further interviews were conducted.

**Table 1:** Respondents’ breakdown by professional category

Category	Number of respondents
National level policy maker in pharmaceutical regulation	1
Senior provincial directors and policy makers	5
Academic in public health	1
Provincial managers of the medicines supply chain	2
Mid-level managers (sub-structure pharmacists; primary healthcare managers)	4
Frontline health workers (clinicians, health promoters, NGO workers)	28
Private sector pharmacists	4
Total	45

#### Non-participant observations

The first author conducted observations on two occasions. The first session was for distribution of HIV treatment and another for distribution of medicines for non-communicable diseases (e.g. diabetes and hypertension). Both sessions took place in Khayelitsha, one of the largest townships in South Africa. During observations, the first author took note of patient-patient and patient-provider interactions and the process in general. Other items that were recorded included the queries that were posed by patients and any information related to patients’ knowledge about their medication.

#### Document review

We reviewed guidelines and standard operating procedures for CBD in order to understand how the models are currently implemented [32, 33].

#### Data analysis

**Fig. 1:**
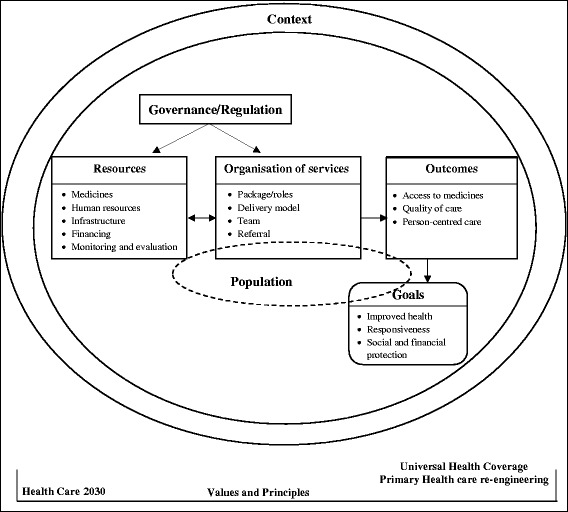
Conceptual framework adapted for this study

The recordings were transcribed verbatim and deductive analysis was applied. We sought for : (a) structure of CBD models, and used the main elements of the *analytical framework of van Olmen* et al. [30], (Fig. 1) which links the central elements required for models to function optimally i.e. *resources* (medicines, human resources, infrastructure, financing, monitoring and evaluation) to the performance of the service delivery platforms. All these elements require good *governance* (policies, regulatory frameworks) and *leadership*, taking into account the population’s needs and demands [34] to attain ATM in terms of its different access dimensions or *outcomes*; i.e.: availability, affordability, accessibility, acceptability and quality) [35, 36] and ultimately improved health status and social and financial protection. Access outcomes can be broadly defined as follows:

-  
*acceptability*: fit between clients and providers’ mutual expectations and appropriateness of care;
-  
*accommodation*: fit between organisation of services and clients’ practical circumstances;
-  
*availability*: fit between existing resources and clients’ needs;
-  
*accessibility*: fit between physical location of healthcare and location of clients;
-  
*affordability:* fit between cost of care and ability to pay [35].

Outcomes are expressed both quantitatively and qualitatively by our adopted framework [30]. However, in the absence of objective outcome- and impact-level data for CBD models, we assessed selected outcomes only qualitatively, from the perspectives of informants. Accessibility is an inherent design feature of CBD models and as such this was not assessed. Using data from interviews and observations, we assessed how models were perceived by informants and patient engagement with CBD services. Our assumption was that if models increase ATM, this could be a proxy for utilisation. In addition, we considered *facilitators and barriers to effective implementation* and *context* factors because CBD models are embedded within a broader health system and these factors can influence outcomes and goals (Fig. 1). Quality was a cross-cutting issue addressing the issues of scientifically and medically appropriate and good quality services. This is determined by aspects such as human resources and good quality medicines.

Data from document review and observations were used to triangulate key informant data.

The first author conducted the initial analysis (coding, retrieving of quotations representative of major themes and interpretation) using Atlas. TI version 7. Emerging themes were discussed with selected key informants through three feedback sessions (participant checking).

## Results

This section starts by presenting an overview of how CBD services are organised (*Typology of CBD models*) then presents the remaining findings according to the elements of the framework (*human resources, medicines, infrastructure* and *population*). Finally, we present our findings related to *governance*, taking into account the implementation context.

### Typology of CBD models

From the interviews with key informants, we found variation in focus and structure of CBD models implemented in the Western Cape Province. Regarding geographical spread, some areas had a single model while others had a combination of models. The mix of models available in an area was primarily dependant on the presence and mandate or interests of particular stakeholders whose activities tended to be geographically demarcated. However, they were all linked to nearby PHC facilities for medicines supply. In this article, we categorised them as formal and informal as explained below:

- I. **Formal:** Models officially recognised and approved by the WCDoH. Services were provided free of charge to the patient. Formally recognised providers were expected to facilitate referrals and linkage to care for patients at risk who require consultation with the health provider. Some models were based on direct involvement of trained HCPs (i.e. nurses and/or post-basic pharmacists), while others were driven by community health workers (CHWs) with some basic health training, linked to NGOs.
- II. **Informal:** Models driven by entrepreneurs with no basic training in health. They charged a service fee to the patient and were not officially recognised by the WCDoH. Informal providers could be described in two ways: either operating under the 'approval' of mid-level management or known anecdotally, but not easily identifiable. The latter operated on a small-scale and could not be easily distinguished from a relative or friend collecting medicines on behalf of the patient. At the time of research, service fees charged by the known informal providers ranged from ZAR10.00–20.00, which was equivalent to approximately US$1.00-2.00. It was unclear how informal providers market their services or initiate services in the absence of the approval of senior provincial leadership.

Patient enrolment in all the CBD models was facilitated by nurses and health promoters during club sessions (group-based education), and patients were asked to provide consent for their information to be supplied to the service provider of their choice. Table 2 shows the range of models that we identified at our study sites. We acknowledge that this list may not be exhaustive for the Cape Town metropolitan area.

**Table 2:** Overview of models for community based distribution of medicines

Type of model	Classification	Human resources	Financing	Beneficiary population as per disease state
1. Distribution in community halls, churches, old-age homes or mobile clinics	Formal	Pharmacist’s assistants, nurses	Health facility budget therefore government funding	HIV and/or NCDs
2. Distribution in small municipal clinics that do not offer NCD services	Formal	Pharmacist’s assistants	Health facility budget therefore government funding	NCDs
3. Home delivery	Informal	Local social entrepreneurs	Out-of-pocket payments	NCDs
4. Home delivery or other community venues^a^	Formal	Community health workers^b^	A few organisations have international funding while the rest receive grants from the Department of Social Development (DSD) and other local businesses.	NCDs

^a^Places where the elderly meet for skills development and social activities, also termed Chronic Disease of Lifestyle clubs which the WCDoH identified through the DSD

^b^Attached to NGOs with service level agreements with the WCDoH

### Resources

#### Human resources

As illustrated in Table 2, task shifting from pharmacists to other HCPs and Non-Health Professionals (NHPs) is a common feature in CBD models. There was contention between participants about the involvement of NHPs and their permitted scope of practice.

Proponents for task-shifting in CBD models argued that this mechanism could address existing human resource shortages in the South African public sector by “de-medicalising” treatment to ensure sustainability of models. Informants cited a situation illustrating lack of sustainability of medicalised models: a clinical nurse practitioner was asked to urgently return to the health facility from a CBD site leaving patients unattended and necessitating their referral back to the health facility.

Another stakeholder (academic) argued that patient counselling by pharmacists, though desired, was in most cases impractical. The informant’s own research showed that pharmacists in the Western Cape spend an average of only three minutes (range: 2–4 min) of face-to-face contact with a patient due to workload pressures. In light of these health workforce issues, stakeholders suggested the need for greater efforts towards empowering patients to manage their own therapy thereby reducing the need for regular contact with HCPs.

Those who opposed involvement of NHPs in CBD cited their lack of accountability to statutory bodies as a major concern in delivering pharmaceutical services. This is currently a grey area in the task-shifting discourse because statutory bodies only regulate personnel who are registered with them.

Other concerns raised by participants related to the capacity of NHPs to: (i) conduct quality assurance (QA) processes (e.g. verifying medicines before handing them over to the patient), (ii) monitor therapeutic outcomes and (iii) link at-risk patients to appropriate care. These tasks are outside their scope of practice therefore, perhaps a more pertinent question is: which tasks should NHPs be expected to carry out? Many informants argued that QA processes should be ensured by the Chronic Dispensing Unit (CDU), a centralised dispensary responsible for dispensing and pre-packing of medicines in the public sector in this province. If performed optimally by ensuring minimal prescribing and dispensing errors, this would eliminate the need for checking parcels at the distribution point upon issue to patients. With QA processes out of the way, this would technically not be a full dispensing process, allowing NHPs to comfortably participate in the process.

It seemed even pharmacists who were responsible for checking the pre-packed medicine packages felt that the QA demands were time consuming and detracted from the intended benefits of both the CDU (which was established to reduce pharmacists’ workload) and of CBD (which was established to take the pressure off health facilities and to improve access for patients).

While some informants mentioned that they would feel comfortable relying on CHWs to issue medicines that were already checked at the CDU, some clinicians were still reluctant. They suggested that CBD activities be placed under the responsibility of registered pharmacy mid-level workers known as pharmacist’s assistants (PAs) as opposed to CHWs. A further suggestion was engagement of private-sector pharmacies to distribute public-sector medicines. In subsequent years, this model was proposed under the NHI scheme [37].

#### Medicines supply management

Our findings show that inefficiencies in procurement (a macro-level issue) affected medicines availability at the CDU where dispensing for CBD programmes takes place. As such, medicines omitted from parcels would require manual dispensing at health facilities, another reason why informants were sceptical about NHPs involvement as the final link to patients. As stated by a senior manager:

*“I wouldn’t like at this stage for community health workers to give medication, because, once in a while, something is missing, because of the out-of-stock situation. Now we got a serious situation as well…the Cape Medical Depot cannot always supply because of change of tender.”*

Another contentious issue raised was the handling and storage of “non-collected” medicines, i.e. parcels not collected by the patient on the appointment date. The handling of medicines by untrained personnel and their storage in transient unregistered sites casts doubt on the integrity of non-collected medicines and as such, these medicines are usually disposed of with resultant cost implications. Informants were of the opinion that some of these risks could be obviated if sites met minimum standards for medicine storage.

#### Infrastructure and logistics

Securing reliable venues for CBD activities emerged as another important aspect of CBD. During the time of our study, services were interrupted at one site because it was no longer available for CBD. The PA at the site expressed concern about the potential loss of confidence by patients experiencing service disruption. In addition to securing venues, opening times for the venues needed careful consideration. This often called for negotiation with the owners of the venue to ensure that times were suitable for the patients.

Reliable transportation for medicine delivery to CBD sites was also identified as a need. Government vehicles could be requested by PAs linked to formal CBD models, but this transportation mode was not accessible to CHWs who often walked to sites and carried the supplies. According to informants, the latter not only posed security risks and environmental risks for the medicines, but created inefficiencies for CHWs with home-based care duties who were often late for CBD activities. Informal providers used bicycles and this was also feared to potentially render medicines vulnerable to environmental risks.

### Outcomes

#### Acceptability of CBD models and accommodation to clients’ practical circumstances

We used our observation data related to patient-provider and patient-patient interactions during the CBD process to look into the *acceptability* of the models. Interactions between patients and providers and between patients were largely positive. Patients showed no restraint in engaging with the providers involved in CBD (both HPs and NHPs) even when they presented late for their appointments. In some cases, CHWs reported taking the initiative to deliver medicines to patients’ homes when they failed to collect at community venues, a means of *accommodating* patients’ practical circumstances. These deviations from formal processes were merely acts of goodwill facilitated by positive patient-provider relationships, but were noted to contribute to *acceptability*. Furthermore, CHWs reported using cost-effective social media methods such as the instant messaging application “WhatsApp” to remind patients of their appointments and to follow-up with those who missed appointments. In this regard, the close patient-provider interactions allowed for some degree of patient follow-up where there had been limited to no follow-up mechanisms in the health system. These experiences also reveal a form of grassroots innovation that could improve patient retention-in-care in the long-run.

Informants collectively felt that CBD models are suitable for patients who are empowered to take responsibility for the management of their illness. From observation during CBD operations, some patients were able to accurately identify their medicines, including identifying any missing medicines when there were medicines availability challenges.

Despite the positive aforementioned aspects, there were some concerns with stigma. At one site (a small municipal clinic which traditionally offered HIV services and was later also used as a distribution site for NCD medicines), patients on ART raised concerns about privacy because their appointments overlapped with patients enrolled in NCD programmes. With medicine collection points for ART being distinct, patients with HIV were easily identifiable and this was a huge concern for those who had not disclosed to family and friends. This raised questions about the appropriateness of integrating HIV and NCDs in the design of CBD models.

At a second site, providers also noticed similar reluctance from clients on ART. The pharmacist’s assistant in charge of CBD at the site said:

*“…we told them that it’s only them who are going there; there are a lot of offices so no one will know why you are walking through that building, what you are going to do there…”*

While in principle, patients should be offered the choice to collect medicines at CBD sites or at the health facility, in practice there seemed to be pressure to enrol all patients onto CBD models, because of the perceived benefits for both the health system and the patients. Asked if patients had a choice regarding their collection point, one PA stated *“…we don’t prioritise that freedom”.* In their view, once patients experienced the benefits of CBD, they appreciated the system and in most cases were no longer interested in the facility-based model.

#### Affordability to patients: to pay or not to pay for CBD services?

As stated earlier, the critical difference between the formal and informal CBD models was that the former provides services at no charge to the patient while the latter levies a user-fee. Many stakeholders grappled with the issue of paid services: some senior managers expressed disapproval of imposing out-of-pocket payments on the premise that medication was free and no direct charges should be introduced to the patients, while others feared that the absence of regulation on levying fees could result in patient exploitation. Indeed, some patients had apparently mentioned to informants that the services were expensive for them but some HCPs still argued that paid services were demand-driven and that many patients were willing to pay for a service offering convenience. One nurse and PA were of the opinion that the elderly derived particular benefits since they often have impaired mobility, lack family and other support to collect medicine on their behalf and many stayed in areas that are not served by formal models. Also, the formal models had limited capacity to serve a large population. Some respondents felt that paid services offset the usual indirect costs for transport fees to the health facility and thus had no objection to charging fees for CBD services.

At the time of our study, one of the four study sites had no history of “fee-for” services, a second site still charged a fee and the remaining two sites had been mandated to cease services that attached a fee. Although some frontline HCPs approved of services levied for a fee at the second site, senior managers had strong reservations. However, HCPs reported that some patients still enquired about the service and attributed increased non-collection of medicines to the management’s decision to stop these paid services. One pharmacist elaborated as follows:

*“A few years ago, we had a courier service that was privately run and we had an objection from the government that it’s unconstitutional to charge patients from a primary health care level. Then we stopped it. The patients benefited a lot from it and up to this day, patients are still asking “When is it coming back and why can’t we have it back?”, because they were prepared to pay. But the department said it is criminal for patients who can’t afford the service. It didn’t make sense to us but it came from the top level to be stopped basically, but it was working well and we were pushing almost 200 parcels a day from the facility.” [Pharmacist]*

In essence, views on paid services were quite divergent, with provincial managers expressing the need to safeguard patients against exploitation and with some frontline HCPs indicating that paid for services are demand-driven and should remain an option to patients.

### Governance: Policy and regulatory issues

As stated earlier, for any service delivery model to function effectively, all health system elements require good governance in the form of policies and regulatory frameworks which consider the population’s needs and demands.

At the time of this study, there was no policy to institute CBD models and guide the implementation efforts in the Western Cape. Stakeholders were not aware of policies in other parts of the world enabling the use of non-registered sites for distribution of medicines for chronic diseases and as an interim measure, they developed standard operating procedures (SOPs), based loosely on available pharmacy and health regulations. Stakeholder views on these SOPs varied. As one provincial manager explained:

*“… this (CBD) is new … There was, like, really no definite law to guide the Pharmacy Council. So, whatever has happened has been an interpretation of the law by someone (provincial stakeholders) …”*

We were informed by a key actor during this study that some engagement between provincial and national stakeholders responsible for the policy making process had commenced by 2014. The South African Pharmacy Council (SAPC), which is the statutory professional body for pharmacy, together with the National Department of Health (NDoH) which has oversight of health activities and legislation, were cited as the two governance bodies responsible for drafting legislation. While recognising that CBD policy development is a national priority and that the process of policy-making can be slow, stakeholders insinuated that the process has not been altogether transparent. We found there was limited consultation of frontline HCPs on the issue and that no feedback on progress of the policy development process was given at this level. One senior manager had some information on the process and reported that a task team had been set-up and was steadily working on developing the policy.

### Stakeholder perspectives on the future CBD policy

In general, informants envisaged that the policy will define organisation of CBD services to ensure the delivery of quality pharmaceutical services as defined by the Good Pharmacy Practice (GPP) standards [38]. There were some shared concerns that some aspects inherent to CBD models do not meet GPP standards, *inter alia*, medicines handling and storage and possible lack of patient counselling.

Some stakeholders justified the current structure of CBD services while others showed disfavour towards some aspects of CBD and offered suggestions for improved organisation and structure. Despite varying opinions on what the content of the CBD policy ought to be, a critical issue that was raised was the need for the policy to be *context specific and pragmatic*. There were concerns that existing CBD models could be jeopardised if the upcoming policy prescribed the use of qualified personnel (HCPs) and/or distribution from health sites only. It must be understood, however, that the call for flexibility is not akin to accepting sub-standard service. Rather, it is a call to be realistic regarding what is both *feasible and sustainable* in the local context. As one manager said:

“…*they (regulators) need to draft the legislation to reflect pharmaceutical services as they are delivered in 2015, and going forward not in 20 years ago and in 30 years ago. Medicines are not ordinary commodities. The integrity of the medicine has to be maintained… but our request collectively to council (SAPC) has been could one look at a framework where one could legally issue the medicines that are not on a health site… and have a set of norms and standards for the issuing of medicines … as long as rules and standards are met and maintained and monitored.”*

Furthermore, the call for regulators’ flexibility stemmed from the simple realisation that more diversification is required if the province (and indeed the country at large) is to truly expand ATM. A private-sector pharmacist elaborated on this aspect as follows:

*“I said to somebody from council (SAPC): we are trying to put down first world standards which is very noble, but we are a resource lacking third world, essentially, third world country. We have a component of first world, but ninety per cent is third world. We are a developing country. I hope it gets taken into consideration, because I think it’s going to make implementation of a lot of what national health (insurance) wants to do almost impossible to start.”*

The quote above called into question the degree of alignment between the province’s and country’s goals of improving ATM and the focus by professional statutory bodies on what were sometimes perceived as rigid standards. There was also a prevailing view that perhaps professional bodies were minimally consulted in the development of CBD strategies and that subsequent disagreements are arising between policy stakeholders:

*“…how much dialogue is actually happening between national, what national (NDOH) is trying to bring through the National Health Insurance versus what Pharmacy Council (SAPC) is saying and the statutes to everybody in terms of best policy. I don’t think they are on the same page as to what the best practice is. (private-sector pharmacist)*

On a positive note, despite a lack of consensus among stakeholders on certain issues, there was notable commitment from the WCDoH leadership to engage with SAPC and eventually align with the future CBD policy. Stakeholders also anticipated that CBD implementation could eventually cost more than is currently envisaged if provinces have to invest in training personnel and adapting venues to meet requirements for medicine handling and storage for example.

## Discussion

CBD models are regarded as a useful way to improve ATM in the Western Cape Province. In this article, we described a range of formal and informal CBD models present using the framework by van Olmen et al. [30]. The framework enabled us to illustrate how the configuration of the elements in each CBD model could contribute to its effectiveness and furthermore, to illustrate the interconnections between the CBD models and the wider health system elements, indicating how policy decisions on each of these elements will likely affect other elements. For example, the framework argues for the need to recognise patients’ own contributions to their personal well-being [30]. Through our research, we noted demand-driven operations by informal providers in a context where senior managers were opposed to the idea. Some differences between formal and informal models were that the formal models are a health system response and therefore, at least in theory resourced and accountable to the system while informal models are grassroots driven, self-funded and with no accountability mechanisms to the health system. However, both have the same goal of improving ATM.

Another key lesson from the application of the framework is that it is the combination of different health system elements that makes a model work well. For example, a decision on the human resource cadre(s) could influence the structure and operational modalities of CBD models, particularly when task-shifting is introduced and mechanisms for accountability and quality assurance become essential. Table 3 summarises what we identified to be facilitators and barriers associated with each CBD element in its current form, an approach we envisage will inform the policy debate.

**Table 3:** Summary of how CBD elements facilitate or constrain CBD implementation

CBD element		Facilitators	Barriers
Medicines		o Centralised dispensing simplifies distribution process.	o Quality assurance processes must be fulfilled by HCPs prior to “last mile” distribution; o Stock-outs of medicines cripples CBD models; o Non-collected medicines cannot be re-dispensed.
Human resources	Community Health Workers	o Positive, close relationships with patients which can facilitate active follow-up when necessary.	o Not able to conduct quality assurance processes.
	HCPs	o Missing medicines from patient-ready parcels can be dispensed manually by the HCP at the CBD site.	o General shortage of HCPs undermine sustainability of deploying them to CBD sites.
	Informal providers	o Demand-driven therefore likely to suit beneficiary needs.	o No governmental oversight which could lead to financial exploitation of patients; o no accountability to professional statutory body which could compromise quality of pharmaceutical services.
Infrastructure and logistics		o Government vehicles available for transportation of medicines for some models.	o Poor transport systems for CHWs causing delays and posing security and environmental risks to medicines; o Availability of venues not always guaranteed.
Patient (population)’s engagement with CBD models		o Positive patient-patient; patient-provider relationships; o Some patients knowledgeable about their treatment regimen and proactive in addressing medicine-related concerns.	o Stigma associated with HIV still a reality.

Despite increased interest in CBD by stakeholders in the WC, medicines are governed by pharmaceutical policy, therefore, who handles them and how they are handled becomes a matter of regulatory interest. This dimension needs to be carefully navigated to ensure safety of the population. As there is currently no CBD policy, we explored how our findings could inform the development of a future policy, through knowledge of context needs and demands. Through this study, we have brought the voices of frontline HCPs to the policy discussion on ATM. As stated by Gilson & Raphaely, “*Policy actors are not just those officially tasked with policy development; they also include those with concern for particular policy issues or likely to be affected by policy developments…*” [39]. We identify HCPs as such because of their important role at the coal-face of the health services and as such as the actual implementers of policy.

We identified some lessons from this study which could inform the policy development process. First, reaching a consensus requires broad stakeholder consultation as part of the policy development process, which to our knowledge has not yet been conducted in this case. Despite some stakeholders being aware that the policy development process had commenced, we found that consultation and feedback on the progress of the process was not inclusive and that frontline HCPs who are responsible for implementing policies were not involved. Acknowledging that policy processes are in essence political, how much influence actors have might be contingent on their position in the political hierarchy, more than their knowledge and understanding of the issue [40]. Hence in this study, we have sought to elevate the voice of frontline HCPs, who possess knowledge and understanding of grassroots issues. This stakeholder group has been referred to as “street-level bureaucrats”: they are tasked with policy implementation and often have to balance policy demands with the realities of their context [41]. Considering their perspectives during the policy development process could result in more responsive policies. As echoed by Morrow (2015), the process of formulating a pharmaceutical policy is as important as the policy document in ensuring collective ownership [42].

Second, the resistance by some stakeholders to aspects of CBD corroborates findings in other countries. Indeed, previous studies have shown that diversion from traditional ways of delivering pharmaceutical services and task shifting in the pharmaceutical sector in its different forms has in many instances met with resistance [43, 44]. Experiences of resistance to different CBD models were documented in Mozambique with the introduction of self-forming patient groups [19] and in Tanzania with the implementation of community retail drug shops, but this changed with time [45]. In Mozambique, as stakeholders gained knowledge and confidence in the model and the benefits became apparent, endorsement increased [19]. In Tanzania, retail drug shops which are a major source of medicines in rural and underserved areas also initially faced resistance, then catalysed development of policies. Of note, the Tanzania model illustrated that even informal providers can be assisted to comply with regulatory standards [45]. Whether or not this will become the experience of providers in our context, remains to be seen.

### Implications for future research and the policy agenda

The current provincial [32, 33] and national [4] goals for UHC in South Africa include both CBD and a commitment to provision of quality services [46], presenting an opportunity to leverage the existing political window. However, while the need to develop a policy to govern CBD activities in South Africa is evident, it is uncertain what changes the anticipated policy will bring to existing models. As earlier indicated, many of our informants hoped that the introduction of policy will not pose a barrier to further implementation of current CBD models. This has been experienced in other contexts where innovation in community-based services began outside of public regulation [47]. We argue that despite the diverging stakeholder views, CBD must be assessed within the lens of what it is endeavouring to achieve - sustainable ATM. The World Health Organisation (WHO) has recommended in other instances that implementing regulation targeted at innovative models should neither decelerate the speed at which action is already taking place nor usher in restrictions that may have a constraining effect on public health service delivery effort [48]. That said, there is need to conduct accurate assessments of the effectiveness of these models and to ensure that they are implemented in a way that ensures patient safety.

In addition, informal models present an additional set of challenges, i.e. the lack of accountability mechanisms and the potential financial burden on patients caused by paid services. While it is true that there are high poverty levels in this context, the paid option is voluntary. Perhaps the critical question is: “Why do patients choose to pay for medicines delivery when they can get a ‘free’ service?”. Since we did not interview patients paying for this service as part of this study, we can only speculate that the parallel system tends to thrive because there is an opportunity cost related to the informal system, i.e. it offers benefits (e.g. the convenience of not having to take time off work, which could result in a cost of a different kind) that might not be present within the formal ‘free’ system. Future studies could assess whether this system imposes a financial or any other burden on patients. If it does, but has other benefits to patients, the next issue is whether the government can lend support to informal providers so that they operate at a lower or no cost to patients.

Finally, further research is required to identify how CBD models have been implemented in other settings and their cost to health systems. Therefore, as a follow-up study, we have designed a scoping review which aims to obtain systematic evidence about design and implementation of CBD models in low resource settings and hard to reach populations in high income countries. We intend to assess whether the issues raised in this article were identified and if so, how they were or could be managed or overcome.

### Study limitations

A limitation of this study was the adoption of the analytical framework after data collection; therefore, not all components were addressed equally during interviews with stakeholders. This is particularly true for monitoring and evaluation of CBD models, an area that requires attention in future studies. Second, the lack of accurate data on outcomes imposed some limitation.

## Conclusion

Improving medicines delivery is integral to attaining UHC and the introduction of CBD in South Africa is one mechanism to achieve this goal. To achieve the intended benefits of CBD, frontline HCPs should be consulted in policy development and consideration should be given to similar models in other contexts. Further research will seek to contribute towards evidence-based development of policy and service delivery guidelines for CBD activities in South Africa within the frameworks of pharmaceutical policy and practice.

## Abbreviations

ATM: Access to medicines
CHW: Community health worker
DSD: Department of Social Development
HCP: Healthcare provider
NGO: Non-governmental organisation
NHP: Non-health professional
PA: Pharmacists’ assistant
PHC: Primary Healthcare
QA: Quality assurance
SOPs: Standard operating procedures
UHC: Universal Health Coverage
WHO: World Health Organisation
